# Optimization of Ultrasound-Assisted Extraction Conditions Followed by Solid Phase Extraction Fractionation from *Orthosiphon stamineus Benth (Lamiace)* Leaves for Antiproliferative Effect on Prostate Cancer Cells

**DOI:** 10.3390/molecules24224183

**Published:** 2019-11-18

**Authors:** Siti Hasyimah Suhaimi, Rosnani Hasham, Mohamad Khairul Hafiz Idris, Hassan Fahmi Ismail, Nor Hazwani Mohd Ariffin, Fadzilah Adibah Abdul Majid

**Affiliations:** 1 Institute of Bioproduct Development, Universiti Teknologi Malaysia, 81310 Johor Bahru, Johor, Malaysia; sheemasuhaimi@gmail.com (S.H.S.); mhkhairul@gmail.com (M.K.H.I.); norhazwaniariffin@gmail.com (N.H.M.A.); 2 School of Chemical and Energy Engineering, Universiti Teknologi Malaysia, 81310 Johor Bahru, Johor, Malaysia; 3 Institute of Marine Biotechnology, Universiti Malaysia Terengganu, 21030 Kuala Terengganu, Terengganu, Malaysia; h.fahmi@umt.edu.my (H.F.I.); f.adibah@umt.edu.my (F.A.A.M.)

**Keywords:** ultrasound-assisted extraction, central composite design, solid-phase extraction, rosmarinic acid, *Orthospihon stamineus*, prostate cancer, antioxidant activity

## Abstract

Primarily, optimization of ultrasonic-assisted extraction (UAE) conditions of *Orthospihon stamineus* was evaluated and verified using a central composite design (CCD) based on three factors including extraction time (minutes), ultrasound amplitude (A), and solvent concentration (%). The response surface methodology (RSM) was performed to develop an extraction method with maximum yield and high rosmarinic acid content. The optimal UAE conditions were as follows: extraction time 21 min, ultrasound amplitudes 62 A, and solvent composition 70% ethanol in water. The crude extract was further fractionated using solid-phase extraction (SPE), where six sequential fractions that varied in polarity (0–100% Acetonitrile in water) were obtained. Next, the six fractions were evaluated for their antioxidant and anti-cancer properties. This study found that Fraction 2 (F2) contained the highest rosmarinic acid content and showed the strongest antioxidant activity. Additionally, F2 showed an anti-proliferative effect against prostate cancer (DU145) with no harmful effect on normal cells.

## 1. Introduction

Prostate cancer is a lethal disease occuring in men. The development of this disease is influenced by various factors such as androgen level, obesity, chronic inflammation, genetics, age, and ethnicity [1]. Clinical treatments of cancer are available, which include hormonal therapy, chemotherapy, radiation therapy, and chemotherapie drugs [2]. However, natural chemotherapy drugs from herbal plants, which may induce apoptosis in cancer cells, can serve as chemotherapeutic agents [3]. Besides that, natural compounds that have high antioxidants have been demonstrated to have a protective effect against cancer development [4].

*Orthosiphon stamineus* (OS) is a well-known herb in South East Asia belonging to the Lamiaceae family. The leaves of OS have traditionally been used in treating inflammation, eruptive fever, rheumatism, diabetes, and jaundice [5]. Numerous scientific studies have been conducted to explore the antiproliferative effect of OS. Sahib et al. [6] discovered that the methanolic extract of OS could improve the activity of Tamoxifen against human responsive breast cancer cells in vitro. The chloroform extract of OS was found to have an anti-proliferative effect against cancer cell lines such as HeLa cervical adenocarcinoma and K562 chronic myelogenous leukemia cell lines [7]. Al-Suede et al. [8] investigated the effect of OS against human prostate cancer (PC3) in vitro and discovered that OS produced selective toxicity against PC3 and was non-toxic to the normal cell line. A high content of phenolic acids such as rosmarinic acid (RA) and a flavonoid content such as sinensetin, eupatorin, and 3′-hydroxy-5,6,7,4′-tetramethoxyflavone (TMF) were found in the OS leaves [9]. RA was reported to exhibit many therapeutic properties such as antioxidant, anti-microbial, and anti-inflammatory properties [10]. Additionally, eupatorin is a powerful inhibitor for in vitro proliferation in breast cancer [11]. Meanwhile, a study conducted by Dong, et al. [12] found that sinensetin prevented the growth of gastric cancer cells and caused apoptosis.

All of these useful phytochemicals can be extracted through various techniques, from simple maceration to the latest technology of supercritical fluid extraction. Handa et al., [13] defined extraction as a separation of the medicinally active portion of plants using selective solvents through standard procedure. Choosing a suitable solvent system is an essential step to extract plant material. Among the commonly used solvents such as methanol, ethanol, propanol, acetone, and ethyl acetate, it was found that ethanol is safer for human consumption, from a toxicological point of view, and is compatibile with the food system [14]. Meanwhile, Thoo et al. [15] reported that the binary-solvent system is better than the mono-solvent system in the extraction of phenolic compounds. Thus, considering these reasons, the binary solvent system (ethanol–water) was employed for this study.

Ultrasound-assisted extraction (UAE) has gained popularity owing to the ultrasound irradiation that can increase reproducibility, shorten extraction times, reduce solvent consumption, lower energy input, and lower temperature as compared with other extraction methods [16]. The cavitation bubbles from the ultrasonic waves allow greater penetration of the solvent into the plant cell wall, which is strong enough to release the intracellular products of the plant [17]. Additionally, the ultrasound probe gives higher efficiency extraction by focusing on a localized sample zone [18]. Among several extraction parameters employed by UAE, ethanol concentration, extraction time, and amplitude are the most investigated parameters [19,20,21].

Subsequent to the extraction process, extract is usually fractionated into several groups of different properties. According to WHO [22], fractionation is a separation process of complex mixture into smaller fractions to obtain a high amount of the desired target compound. It is known that crude herbal extract is very complex because it has thousands of phytochemicals with various chemical properties [23]. Therefore, fractionating herbal extract can enhance its quality according to its chemical characteristic based on solvent property. Solid-phase extraction (SPE) is one of the fractionation techniques used for the separation of desired compounds from the crude extract. SPE is favorable because the process is fast, and can be viewed as a cost-effective technique because it significantly reduces the usage of solvent compared with the liquid–liquid extraction technique [24]. Furthermore, this technique offered numerous types of sorbent such as reversed-phase, normal-phase, and ion-exchanged sorbent [25].

Nevertheless, to the best of our knowledge, no study has been performed to investigate the effect of different fractions separated from OS leaf extract on the anti-proliferative effect against the prostate cancer cell line. The objective of this study was to identify the active fraction that has anti-cancer properties against in vitro prostate cancer. Initially, prior to fractionation by the SPE technique, bioactive compounds of OS leaves were extracted using the optimized UAE conditions. The optimized conditions for UAE were developed through a response surface methodology (RSM) method based on yield and phytochemical compounds extracted. Subsequently, these fractions were subjected to antioxidant assay and in-vitro anti-cancer assay.

## 2. Results and Discussion

### 2.1. Preliminary Study

#### 2.1.1. Effect of Extraction Time on Total Yield and Yield of Rosmarinic Acid

**Figure 1:**
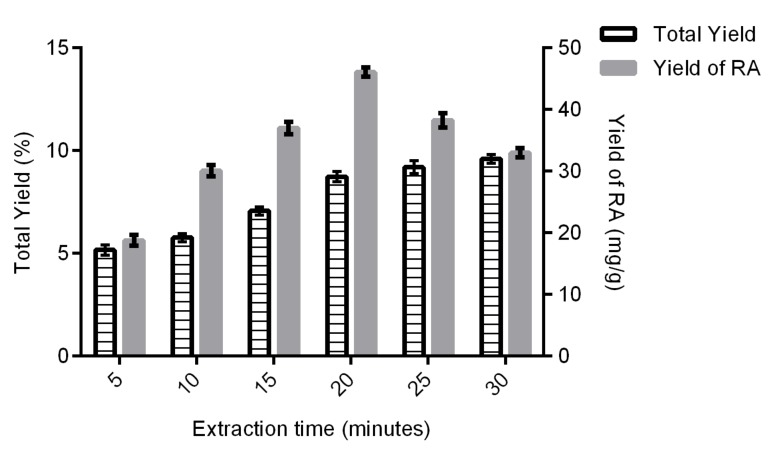
Effect of extraction time on total yield and yield of rosmarinic acid (RA). Data were generated from three experiments (*n* = 3) and presented as mean with standard deviation as error bars.

Extraction time is a crucial parameter in solvent extraction as it can reduce time and save cost. The effects of extraction time on the total yield of OS extract and yield of rosmarinic acid are depicted in Figure 1. An increased extraction time led to an increase in the total yield of OS. However, it was observed that the total yield of OS was not significantly changed after 20 min. This circumstance can be explained using Fick’s second law of diffusion, which predicts that, after a certain time, there will be a final equilibrium between the solute in the solid matrix (plant material) and the bulk solution (extraction solvent) [26]. The yield of rosmarinic acid increased with the increased extraction time from 5 min to 20 min. However, it fell at 25 min onwards. Prolonged extraction time may cause the oxidation of phenolics [27], as well as intensify the loss of solvent through vaporization, and thus may affect the solvent to solid ratio [28]. Therefore, the suitable range for extraction time is from 15 to 25 min.

#### 2.1.2. Effect of Ethanol Concentration on Total Yield and Yield of Rosmarinic Acid

**Figure 2:**
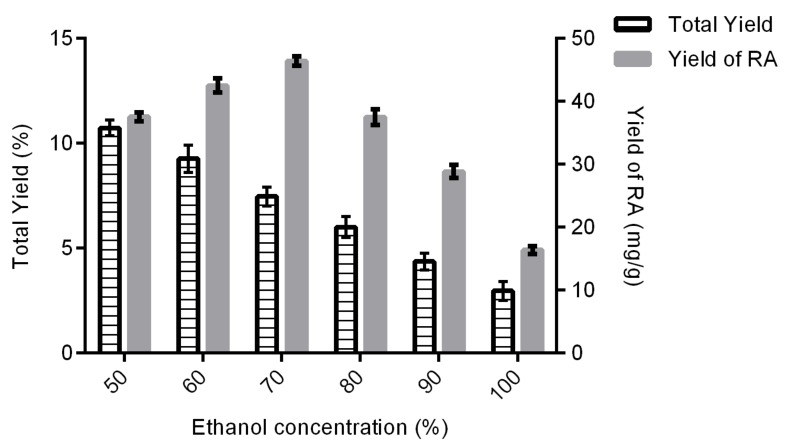
Effect of ethanol concentration on total yield and yield of rosmarinic acid. Experiment was conducted in triplicates (*n* = 3).

According to Figure 2, the total yield of extract was decreased when the concentration of ethanol increased, which might be because of the decrease in solvent polarity; thus, RA, which is a major compound of OS and is a polar compound, was eluted first with a more polar solvent. This contributes to the decrease in total yield. Five hydroxyl groups were found in RA, which is a hydrophilic compound. The hydroxyl groups interact with the solvent system by hydrogen interaction for better solubility [29] (Lau and Chua, 2019). Meanwhile, the yield RA increased with the increasing concentration of ethanol. However, the yield of RA started to decline when the percentage of ethanol was higher than 70%. This circumstance happens because of a lack of water. The presence of water, which acts as a swelling agent, can enhance the extraction efficiency [30] by increasing the contact surface area of the plant material and the solvent [31]. This demonstrates that binary solvent system (ethanol–water) is more efficient compared with a single solvent system (100% ethanol), as the binary solvent system produced a greater yield of RA. Therefore, the range selected for optimization is between 60% and 80% of the ethanol concentration.

#### 2.1.3. Effect of Amplitude on Total Yield and Yield of Rosmarinic Acid

**Figure 3:**
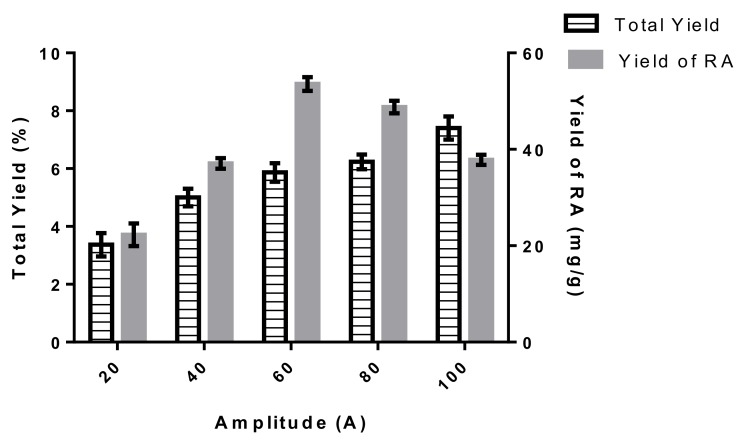
Effect of amplitude on total yield and the yield of rosmarinic acid. Data were generated from three experiments (*n* = 3) and presented as the mean with standard deviation as error bars.

Figure 3 shows the effect of amplitude on the total yield and yield of RA. The main driving force behind UAE is the acoustic cavitation force, which is proficient at inducing a series of compression and rarefactions in the molecules of the solvent, thus causing the formation of bubbles on the surface of the plant matrix [32]. Amplitude indicates the height of the waves. A huge amplitude of the sonication probe means a high intensity of sonication is transmitted to the plant extract [33], which explains the increase of the total yield and yield of rosmarinic acid as the amplitude increased from 20 A to 60 A. Generally, the high amplitude may enhance extraction efficiency, because an increase in amplitude causes an increase in the number of compression and rarefaction cycles of ultrasonic waves. Thus, it causes higher delivery of the compounds [34]. This study is in agreement with research conducted by Dey and Rathod [35], who found that the extraction yield of β-carotene increased as the amplitude of the waves increased. Entezari et al. [36] stated that when amplitude is increased, this causes a large amount of energy to be transferred for the cavitation phenomena to take places. Thus, the cavities form and explode more energetically. This promotes significant penetration of the solvent into the plant matrix and increases the mass transfer rate. However, at 80 A onwards, the yield of rosmarinic acid decreased. A similar trend was observed from the study conducted by Singh et al. [37], who found that when increasing the amplitude over a particular value, the yield was decreased. They concluded that high amplitude generates the formation of bubbles, which in turn hinder the propagation of the pressure waves. Meanwhile, Filgueiras et al. [38] reported that higher amplitude creates a greater number of cavitation bubbles, which might damage more cell walls of the plant. Thus, the optimum range selected for amplitude is 40–80 A.

### 2.2. Optimization of Extraction Conditions OS using Response Surface Methodology

#### 2.2.1. Analysis of Variance (ANOVA)

In order to evaluate the adequacy and fitness of the model, the significance of the regression model, coefficient of determination (R^2^), adjusted R^2^, lack of fit, and coefficient of variation (CV) were used. Generally, a good model must be significant, and the lack of fit must be insignificant. R^2^ is a statistical measurement of the closeness of data to the fitted regression line [39]. Thus, the value of R^2^ should be as close as possible to 1 [18] and R^2^ should be at least 0.80 to ensure a good fit of the model [40]. Adjusted R^2^ is a corrected value of R^2^ after the elimination of the additional model terms. The adjusted R^2^ is considerably smaller than the R^2^ if many non-significant terms were included in the model [41]. Thus, an adjusted R^2^ with a higher value than R^2^ resulted in a more prominent model [42]. The CV measures the expression of standard deviation as a percentage of the mean. A smaller CV indicates better reproducibility. Therefore, a high value of CV indicates an unsatisfactory response model. The value of lack of fit must be non-significant (*p* > 0.05).

The *p*-value, as shown in Table 1 and Table 2, indicates that all models are significant, which is desirable as it shows that the terms in the model have a significant effect on the response. The lack of fit is also insignificant. In this study, a good fit model was obtained for the total yield and yield of rosmarinic acid, with R^2^ values of 0.9726 and 0.9763, respectively, where all the values were close to 1. The CVs for the total yield and yield of rosmarinic acid gave low values, which were 6.27% and 7.49%, respectively, indicating that the experiment was well performed. This means that the model has excellent accuracy and less dispersion. Table 1 and Table 2 summarize the ANOVA analysis for the total yield and yield of rosmarinic acid, respectively.

#### 2.2.2. Effect of Process Variables on Total Yield of Crude Extract

**Figure 4:**
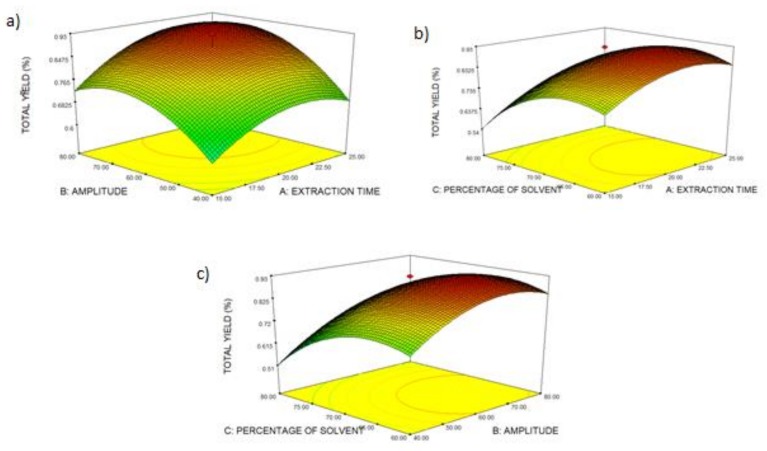
Surface plot of total yield of *Orthosiphon stamineus* (OS) extract: (**a**) extraction time and amplitude, (**b**) extraction time and percentage of solvent, (**c**) amplitude and percentage of solvent.

Figure 4a shows the effect of extraction time (A) and amplitude (B). Both parameters (A and B) give a significant effect on the yield of crude extract. Also, the quadratic (A^2^ and B^2^) was significant. However, the interaction between these parameters (AB) was found to be not significant (*p* > 0.05). The deeper the red color, the higher the total yield, and the highest point represents the maximum total yield produced by the two factors (A and B). The total yield reached the highest at 20 min and 60 A. From the surface plot, a longer extraction time increased and then decreased the yield of extract. This happened because the plant cell is damaged owing to acoustic cavitation by ultrasound as time prolonged [38]. The yield of OS extract increased as the amplitude increased. The higher amplitude of sonication gives a higher intensity of the sonication transmitted to the plant extract, which increases the sonication effect [32]. The effect of extraction time (A) and ethanol concentration (C) on the total yield is shown in Figure 4b. A, C, A^2^, and B^2^ exhibit a significant (*p* < 0.05) effect towards total yield. Meanwhile, the interaction between AB is not significant (*p* > 0.05). An extraction time of 20 min and 70% ethanol concentration contribute to the maximum value of total yield. The solvent is one of the most crucial factors that affect the performance of the extraction process, because solvent will affect the dissolution and diffusion of phytochemicals from plant materials into bulk medium [43]. The graph shows that the total yield of OS extract decreased as the ethanol concentration was further increased from 70% to 100%. The presence of water content creates a greater interaction between the solvent and the internal wall of plant tissue, which increases the yield of extract [44]. Meanwhile, Figure 4c describes the interaction effect between the amplitude (A) and percentage of solvent (B). Both parameters significantly (*p* < 0.05) affect the total yield. The total yield reached the highest when the concentration of ethanol was 70% and the amplitude was 60 A.

#### 2.2.3. Effect of Process Variables on Total Yield of Crude Extract and Yield of Rosmarinic Acid

**Figure 5:**
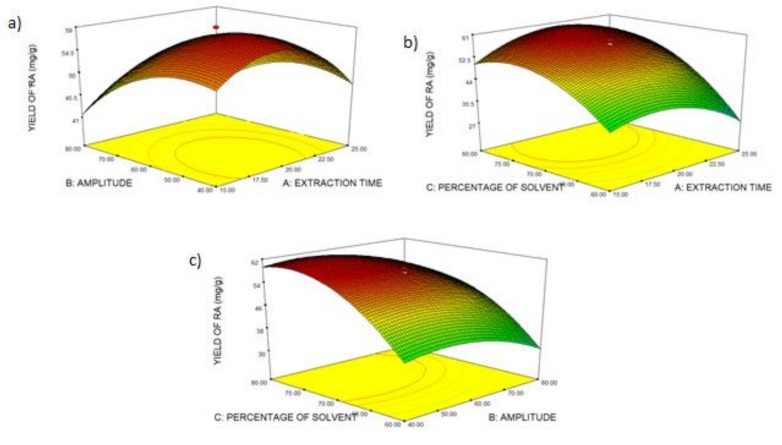
Surface plot of the yield of rosmarinic acid: (**a**) extraction time and amplitude, (**b**) extraction time and percentage of solvent, (**c**) amplitude and percentage of solvent.

Figure 5a shows the three-dimensional surface plot of extraction time (A) and amplitude (B) on the yield of rosmarinic acid. Factor A and AB did not have a significant effect (*p* > 0.05) on the yield of RA, while factor B is significant (*p* < 0.05) towards the yield of RA. The yield of RA reached the maximum value at an extraction time of 20 min and amplitude of 60 A. Figure 5b shows the effect of extraction time (A) and percentage of solvent (C) on the yield of RA. The interaction between A and C (AC) is significant. At 20 min of extraction time and 70% ethanol concentration, the yield of RA is at its maximum value. Figure 5c describes the effect of factor B and C. The linear (B and C) and quadratic (B^2^ and C^2^) effects on the yield of RA were significant. However, the interaction (BC) was not significant. When amplitude, extraction time, and percentage of solvent increase, the yield of RA increases. A longer extraction time aids the disruption of the cell walls and the release of rosmarinic acid into the solvent. Amplitude represents the height of the waves, which plays an essential role in the intensification of extraction. Increasing the amplitude leads to an increase in the number of compression and rarefaction cycles of ultrasonic waves. Thus, there is a higher delivery of the compound [32]. However, as they keep increasing, the yield of RA decreases. The value of RA decreased because of the sensitivity of RA towards the ultrasonic amplitude power. This phenomenon occurred because the bubbles collapse more violently at a high amplitude of ultrasonic waves [45]. RA is a major compound in OS and is a phenolic compound. Ethanol and water were used in this study as a binary solvent system because it was reported that a binary solvent or multiple solvent system gives a higher yield of phenolic compounds than a single solvent system [24]. There is also a study reporting that a portion of water is vital in the extraction solvent to increase the diffusion of phenolic compounds through plant cells [46]. The yield of RA increased rapidly at 60% to 70% of ethanol and decreased gradually with the increasing percentage of solvent. This might be owing to the decrease in the polarity of the solvent as rosmarinic acid is a polar compound. Following the principle of “like dissolve like”, solvents would only extract those compounds that have a similar polarity to the solvents [47].

#### 2.2.4. The Optimal Condition and Validation of Model

The optimization of OS using UAE was completed using CCD. In this study, the optimization was carried out to achieve the maximum yield of OS extract and yield of rosmarinic acid. Table 3 shows the optimum condition given by the software.

Validation of the model was performed (Table 4) to prove the capability of RSM through the predicted value given by the Design-Expert software.

### 2.3. Fractionation of OS by Solid Phase Extraction (SPE)

**Figure 6:**
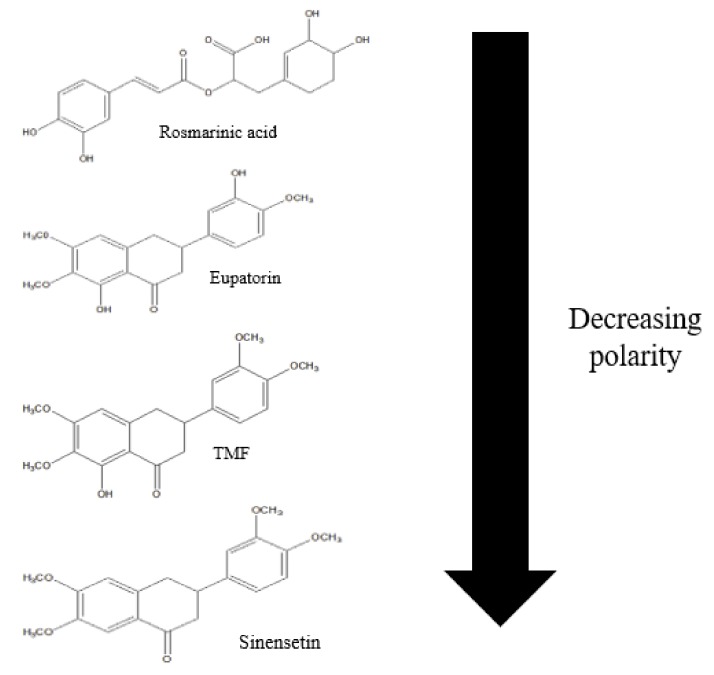
Polarity of the markers of OS. TMF, 3′-hydroxy-5,6,7,4′-tetramethoxyflavone.

A reversed-phase column, C18, was employed for fractionation of OS using acetonitrile–water at different concentrations (0–100%) as the elution solvent system. The crude extract of OS and its various fractions were investigated by HPLC. Rosmarinic acid, eupatorin, TMF, and sinensetin were used as active analytical marker and their polarities are summarized in Figure 6. Total analytical marker content of each fraction was calculated based on the result obtained from HPLC analysis (Equation (1)).

(1) $$\left(\frac{w}{w}\right)\%\;=\;\frac{Concentration\;\left(ppm\right)\times 0.01}{\left(Solid\;content\;\left(g\right)\times 1000\right)\times 100}$$

Rosmarinic acid, which is the most polar compound, was eluted in the first three fractions, Fraction 1 (F1), Fraction 2 (F2), and Fraction 3 (F3). C18 is known as a reversed-phase column where the stationary phase is non-polar and the mobile phase is polar [48]. The non-polar stationary phase retained the non-polar compound, hence most polar compounds elute first. This is because of the Van Der Waals interaction forces between the sorbent, mobile phase, and compound of the sample [49]. As the percentage of acetonitrile increases, the polarity of the elution solvent is reduced. Thus, this explains why Fraction 4 (F4) and Fraction 5 (F5) contained no rosmarinic acid. According to Table 5, the present study shows that rosmarinic acid is most abundant in F2 (148.62 ± 0.46 mg/g), eupatorin, (114.24 ± 0.40 mg/g), TMF (89.88 ± 0.45 mg/g), and sinensetin (45.88 ± 0.40 mg/g) quantities are the highest in F4. The total content of rosmarinic acid was increased from 196.14 mg/g of the crude extract to 216.66 mg/g of the total fraction. Eupatorin was increased from 168.54 mg/g of crude extract to 188.61 mg/g of total fractions, while TMF was increased from 11.38 mg/g of crude extract to 117.06 mg/g, and lastly, sinensetin was increased from 18.87 mg/g of the crude extract to 45.88 mg/g of total fractions. This study shows that fractionation of the crude extract enhanced the concentration of the compounds. A similar trend was found by research conducted by Chua et. al. [50], who found that there is an increment in concentration of Rutin from *Labisia Pumila var. Alata* extract after the fractionation process was applied.

### 2.4. Antioxidant Activity

An antioxidant is known as any substance that can delay oxidative damage to the target molecule. Plant structure is rich with phenolic compounds and polyphenols. Normally, these antioxidant compounds are in the phenolic form [51]. ABTS assay was used because it is rapid, robust, and accurate for analyzing the antioxidant capacity of extracts and plant materials. However, the use of at least two methods for determining antioxidant properties was also considered, as suggested by Schlesier et al. [52]. Thus, in this study, ABTS and DPPH assays were chosen because they are more accurate and reliable than the FRAP assay for assessing antioxidant capacities of plant extracts [53].

**Figure 7:**
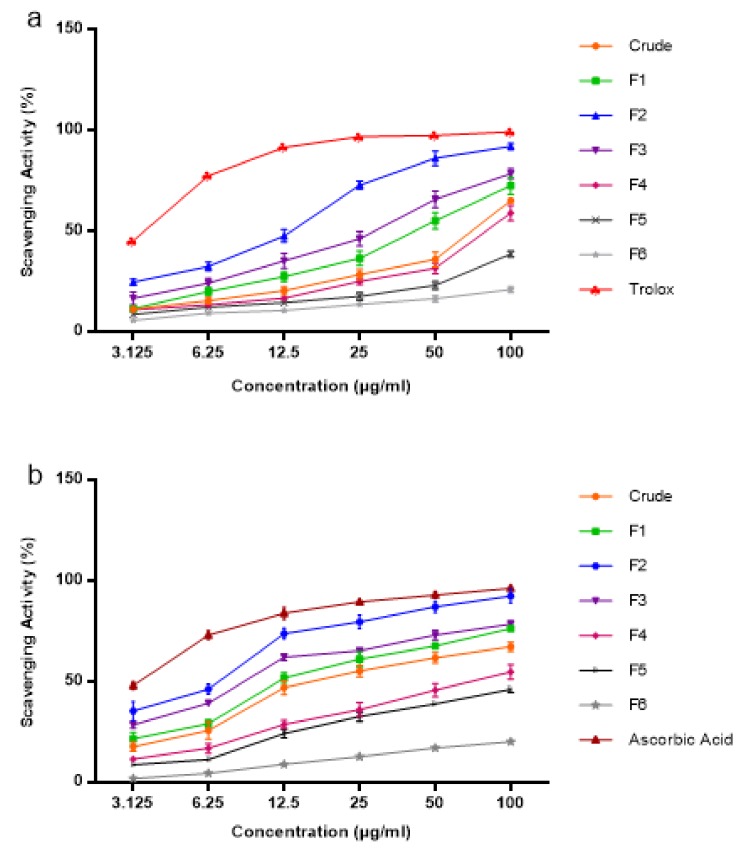
Activity of OS and its fractions in two different assays: (**a**) 2,2′-Azino-bis(3-Ethylbenzothiazoline-6-sulphonic acid) (ABTS) scavenging activity (%) and (**b**) 2,2-Diphenyl-1-picrylhydrazyl (DPPH) scavenging activity (%). Each data represent the mean (*n* = 3) with standard deviation.

The results of ABTS and DPPH radical scavenging are shown in Table 6 and Figure 7. The radical scavenging was observed at different concentrations of 3.125 µg/mL, 6.25 µg/mL, 12.5 µg/mL, 25 µg/mL, 50 µg/mL, and 100 µg/mL, which was prepared by serial dilution. Overall, F2 was revealed to possess the best antioxidant properties, which led to more potent radical scavenging effects, mainly associated with the high content of phenolic compound. Fraction F2 was found to have the highest rosmarinic acid compared with other fractions and crude. Moreover, RA has more hydroxyl groups compared with eupatorin, TMF, and sinensetin. Pavithra et al. [54] reported that free radical scavenging activity is highly related to the availability of a hydroxyl group in the phenolic compound. The phenolic hydroxyl group is a good hydrogen donor that reacts with radical species, halting the cycle of radicals’ productions [55]. Ho et al. [56] reported that radical scavenging of OS is related to their high content of rosmarinic acid. F5 and F6 were found to have no 50% radical scavenging capacity (IC_50_).

### 2.5. Antiproliferative Effect of OS and Its Fractions

**Figure 8:**
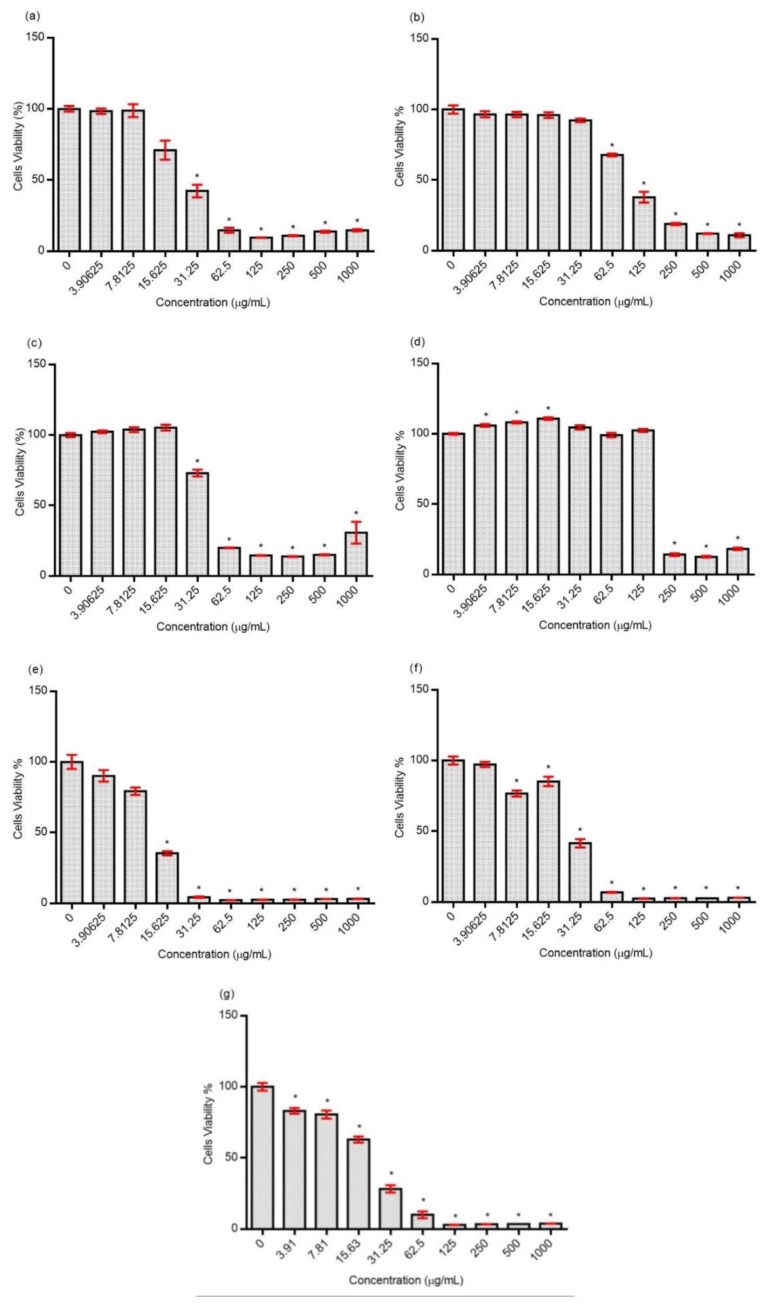
Antiproliferative effect of OS and its fractions on DU145 cell line after 24 h of treatment. (**a**) Crude extract, (**b**) F1, (**c**) F2, (**d**), F3, (**e**) F4, (**f**) F5, and (**g**) F6. The data represent the mean ± SD of three experiments. * represents significant results (*p* < 0.05) compared with control (untreated DU145 cells).

The antiproliferative effect of different fractions of OS on prostate cancer (DU145) is shown in Figure 8. The crude extract of OS and F2 significantly decreased the cell viability at a concentration of 31.25 µg/mL to 1000 µg/mL. F1 significantly reduced the cell viability at a concentration ranging from 62.25 to 1000 µg/mL. As for F3, the cell viability decreased substantially at a concentration of 250 to 1000 µg/mL. F4 was found to significantly reduce cell viability at a concentration between 15.63 and 1000 µg/mL. F5 exhibited a significant anti-proliferative effect at a concentration of 7.81 µg/mL until 1000 µg/mL and, lastly, F6 showed a remarkable decreased in cell viability at a concentration of 3.81 µg/mL to 1000 µg/mL.

**Figure 9:**
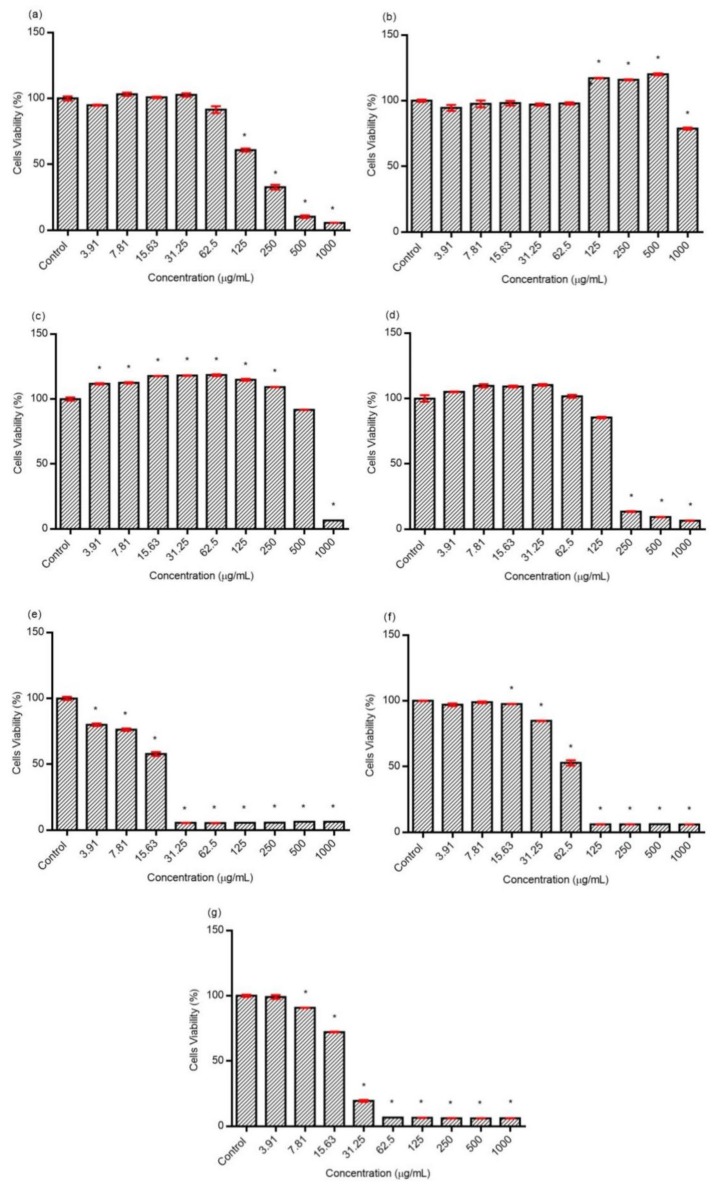
Cytotoxicity analysis of OS and its fractions on the HSF 1184 cell line after 24 h of treatment. (**a**) Crude extract, (**b**) F1, (**c**) F2, (**d**), F3, (**e**) F4, (**f**) F5, and (**g**) F6. The data represent the mean ± SD of three experiments. * represents significant results (*p* < 0.05) compared with the control (untreated HSF 1184 cells).

In order to determine the toxicity of crude OS and its fractions, a toxicity study on a normal cell line, human skin fibroblast (HSF 1184), was conducted. Figure 9 depicts the cytotoxic effect of the samples. The IC_50_ values of samples in both DU145 and HSF 1184 are shown in Table 7. Among all fractions, F4 shows a more pronounced antiproliferative effect against DU145 cell line with an IC_50_ of 12.38 µg/mL. However, F4 exhibits the lowest IC_50_ value (14.47 µg/mL) compared with other fractions on a normal cell line (HSF 1184), which suggests that F4 gives an adverse toxic effect on the normal cell line. As stated by Galati and O’Brien [57], an effective anti-cancer agent must cause no harmful effects on the normal cell line. Therefore, this study found that F2 exhibited a promising anti-cancer property by considering the IC_50_ from both cell lines (DU145 and HSF 1184). Additionally, our finding is in agreement with a study conducted by Al-Suede et al. [8], in the aspect of the anti-cancer potential of OS. They reported that OS extract, using the supercritical fluid extraction technique, exhibits an anti-cancer property on the PC3 prostate cancer cell line. Concurrently, our result also showed potent anti-cancer activity on the DU145 prostate cancer cell line.

As discussed in Section 3.3, F2 is rich in rosmarinic acid. Rosmarinic acid is a phenolic compound that has five hydroxyl groups. Several studies have investigated the therapeutic potential of rosmarinic acid such as anti-cancer activity both in vitro and in vivo. Xavier et al. [58] found that rosmarinic acid significantly inhibits the action of the COX-2 pathway, consequently preventing cancer. Han et al. [59] discovered that rosmarinic acid is a potential therapeutic agent against colorectal cancer. Recently, rosmarinic acid has been reported to have an anti-proliferative effect against prostate cancer cell lines [60].

## 3. Materials and Methods

### 3.1. Materials

The powdered form of OS was purchased from a local supplier (Ethno Resources, Sungai Buloh, Selangor Malaysia) with a particle size of 40 meshes and stored at room temperature in a dark environment. Dulbecco’s modified Eagle’s medium (DMEM), fetal bovine serum (FBS), penicillin strep (PS), and trypsin were purchased from Gibco, Life Technologies (Rockville, MD, USA). Ethanol, acetonitrile, methanol, formic acid, and dimethyl sulfoxide (DMSO) were purchased from QRec Asia (Selangor, Malaysia). 2,2-Diphenyl-1-picrylhydrazyl (DPPH), 2,2’-Azino-bis(3-Ethylbenzothiazoline-6-sulphonic acid) (ABTS), standard rosmarinic acid, eupatorin, sinensetin, and 3′-hydroxy-5,6,7,4′-tetramethoxyflavone were acquired from Sigma-Aldrich, Taufkirchen, Germany. Trolox and ascorbic acid were purchased from Merck, Darmstadt Germany.

### 3.2. Ultrasound-Assisted Extraction (UAE)

UAE was performed using a sonicator (Fisher Scientific, Waltham, MA, USA). A quantity of 3 g of dried OS was extracted at a particular ethanol concentration (50–100%), amplitude (20–100 A), and extraction time (5–30 min). The pulse on and pulse off was set to 5 min to reduce electrical consumption and prevent overheating. The extract was filtered, dried in an oven (45 °C), and weighted before being stored at 4 °C.

### 3.3. Total Yield

The average percentage of yield was calculated using Equation (2):

(2) $$Total\;yield=\frac{W_{1}}{W_{0}}\;\times 100,$$

where W_1_ is sample weight and W_0_ is weight measured before extraction.

### 3.4. High-Performance Liquid Chromatography (HPLC)

Quantification of the selected phytochemicals by HPLC was conducted according to Saidan et al. [61] with slight modifications. A C18 column (Phenomenex, Torrance, CA, USA) was used as a stationary phase, while the mobile phase was 0.1% formic acid solution (A) and acetonitrile (B) utilizing a gradient elution system, as depicted in Table 8. The flow rate was set at 1 mL/min with 0.005 mL injection volume and 18 min separation time. The wavelength was set at 325 nm and data were analyzed by Empower 3 software (Waters).

### 3.5. Experimental Design

Response surface methodology (RSM) was carried out to develop a second-order polynomial model for total yield and yield of rosmarinic acid, respectively. Design-Expert Software package (version 7.1.5, State-Ease Inc., Minneapolis, MN, USA) was utilized in this study. Central composite design (CCD) was employed as a tool to optimize the OS extraction condition parameter using UAE. The three independent variables were sonication time (X_1_), amplitude (X_2_), and ethanol concentration (X_3_), while the dependent variables were total yield (Y_1_) and yield of rosmarinic acid (Y_2_). A total of 17 runs and 3 center points were generated according to the software. The level of extraction parameters (independent variables) was obtained from the preliminary study conducted. Table 9 exhibits the level of extraction parameters.

### 3.6. Verification of the Models

The optimal conditions for the extraction of OS were obtained using CCD. The experimental and predicted values of the total yield of OS and yield of rosmarinic acid were compared in order to determine the validity of the model. The percentage error was calculated using Equation (3). The experiment was carried out in triplicate to ensure the accuracy of the model.

(3) $$Error\;\left(\%\right)\;=\;\frac{Experimental\;value-Predicted\;value}{Predicted\;value}\;\times 100$$

### 3.7. Solid-Phase Extraction (SPE)

The SPE process was conducted following the method from Lau et al. [24], with slight modifications. Chromabond C18 SPE cartridge (6 mL/1000 mg) was fixed to the port of the SPE vacuum manifold. First, the cartridge was conditioned using methanol (12 mL) and then equilibrated by 6 mL of 0.5% formic acid in water. Then, 1 mL of crude extract, which was dissolved in 60% methanol, was loaded onto the SPE column. Next, SPE was conducted using gradient elution. In this study, a bi-solvent system (water-acetonitrile) was utilized. Table 10 summarizes the elution solvent used.

### 3.8. Antioxidant Activities

#### 3.8.1. ABTS Assay

ABTS radical scavenging activity was evaluated according to Re et al. [62], with slight modifications. ABTS solution was prepared in water at 7 mM from its powder. Next, ABTS radical was produced by reacting ABTS stock solution with 2.45 mM potassium persulfate. The mixture was incubated in the dark, at room temperature, for 12–16 h. After incubation, the ABTS radicals were diluted with methanol until the absorbance reading was 0.7 ± 0.1 at 750 nm. Subsequently, the reaction mixture was prepared by adding 100 µL of ABTS radicals with 100 µL of the samples. This was followed by incubation at room temperature for 6 min. Afterwards, the optical density of the reaction mixture was measured at 750 nm using ELx800 Absorbance Microplate Reader (Biotek Instrument, Winooski, VT, USA). Trolox (3.125–100 µg/mL) was used as the positive control, while ABTS solution in the absence of the sample was used as the negative control.

#### 3.8.2. DPPH Assay

Scavenging activity of DPPH radical was conducted according to Brand-Williams et al., [63] with slight modifications. DPPH solution was prepared in methanol at 0.2 mM. Then, 100 µL samples were mixed with 100 µL of DPPH solution. The sample mixture was kept at room temperature in a dark place for 30 min. The absorbance was measured at 515 nm using ELx800 Absorbance Microplate Reader (Biotek Instrument, USA). Ascorbic acid (3.125–100 µg/mL) was used as a standard. The negative control for this analysis was the DPPH solution in the absence of sample. The percentage of free radical scavenging for both the DPPH and ABTS radical scavenging assay was calculated using Equation (4).

(4) $$Radical\;scavenging\;activity\;\left(\%\right)=\left[\;1-\frac{Absorbance\;of\;sample}{Absorbance\;of\;control}\;\right]\times 100$$

### 3.9. Cell Culture Maintenance

Prostate cancer cell line (DU145) and normal fibroblast cell line (HSF 1184) were purchased from American Type Culture Collection (ATCC), Rockville, USA. The cells were maintained in DMEM supplemented by 10% FBS and 1% PS.

### 3.10. Antiproliferation Study

Antiproliferation study was conducted via MTT assay (Mosmann, 1983). Galati and O’Brien [57] stated that the acceptable and potent anti-cancer agent should be non-toxic to normal cells. In this study, the analyses were performed on both prostate cancer cells (DU145) and normal fibroblast cells (HSF 1184). Cells were seeded at density 5 × 10^3^ cells per well and incubated overnight. Treatment was initiated by adding extracts at a concentration ranging from 0 to 1000 µg/mL. After 24 h of incubation, MTT solution was added and incubated for 4 h. Developed MTT formazan was then dissolved in DMSO and measured at 570 nm.

### 3.11. Statistical Analysis

All data were presented as mean ± standard deviation and all tests were done at least in triplicate. The statistical significance was conducted using GraphPad Prism 6.0 (GraphPad Software, Inc., San Diego, CA, USA) and analysis of variance (ANOVA) was conducted using design expert software version 7.1.5 (STAT-EASE Inc., Minneapolis, MN, USA).

## 4. Conclusions

UAE was found to have a significant effect on the yield and rosmarinic acid from OS leaves. CCD was employed to optimize the extraction condition of OS leaves. The optimum point predicted by the software was at 21 min of extraction time, 62 A amplitude, and 70% ethanol. F2, which has the highest RA content (148.62 ± 0.46 mg/g), was selected as the most potent fraction that exhibits significant antiproliferative activity against DU145 prostate cancer line, and offered no adverse effect on the normal cell line. Also, it shows the lowest IC_50_ in the ABTS (11.29 µg/mL) and DPPH (5.975 µg/mL) radical scavenging assays.

## Figures and Tables

**Table 1:** Analysis of variance (ANOVA) for the total yield. A: extraction time, B: amplitude, C: ethanol concentration.

Source	Sum of Square	Degree of Freedom	Mean Square	F-Value	*p*-Value (prob > F)	Significance
Total Yield (R^2^ = 0.9726)						
Model	0.44	9	0.049	27.59	0.0001	Significant
A	0.053	1	0.053	29.78	0.0009	
B	0.088	1	0.088	49.44	0.0002	
C	0.11	1	0.11	61.68	0.0001	
AB	2.379 × 10^−3^	1	2.379 × 10^−3^	1.34	0.2846	
AC	1.605 × 10^−3^	1	1.605 × 10^−3^	0.91	0.3730	
BC	9.625 × 10^−4^	1	9.625 × 10^−4^	0.54	0.4851	
A^2^	0.061	1	0.061	34.53	0.0006	
B^2^	0.094	1	0.094	53.29	0.0002	
C^2^	0.13	1	0.13	73.92	<0.0001	
Residual	0.012	7	1.773 × 10^−3^			
Lack of fit	0.010	5	2.064 × 10^−3^	1.88	0.3824	Not significant
Pure error	2.175 × 10^−3^	2	1.088 × 10^−3^			

**Table 2:** Analysis of variance (ANOVA) for the yield of rosmarinic acid (RA). A: extraction time, B: amplitude, C: ethanol concentration.

Source	Sum of Square	Degree of Freedom	Mean Square	F-Value	*p*-Value	Significance
Yield of Rosmarinic Acid (R^2^ = 0.9763)						
Model	2807.11	9	311.90	32.10	0.0001	Significant
A	17.11	1	17.11	1.76	0.2261	
B	251.19	1	251.19	25.86	0.0014	
C	1372.34	1	1372.34	141.26	<0.0001	
AB	13.13	1	13.13	1.35	0.2831	
AC	95.50	1	95.50	9.83	0.0165	
BC	1.31	1	1.31	0.13	0.7243	
A^2^	552.74	1	552.74	56.89	0.0001	
B^2^	252.92	1	252.92	26.03	0.0014	
C^2^	781.62	1	781.62	80.45	<0.0001	
Residual	68.01	7	9.72			
Lack of fit	65.37	5	13.07	9.92	0.0941	Not significant
Pure error	2.64	2	1.32			

**Table 3:** Optimum conditions of the extraction.

Independent Variables			Dependent Variables	
Extraction time (minutes)	Amplitude (A)	Percentage of ethanol in water (%)	Total yield (%)	Yield of rosmarinic acid (mg/g)
21 min	62	71%	0.908	56.48

**Table 4:** Validation between predicted value and experimental value.

Responses	Predicted Value	Experimental Value	% Error
Total yield (%)	0.908	0.872	3.96
Yield of rosmarinic acid (mg/g)	56.48	59.1	4.43

**Table 5:** Concentration of biomarkers collected for each fraction and crude extract. TMF, 3′-hydroxy-5,6,7,4′-tetramethoxyflavone; OS, *Orthosiphon stamineus*.

Samples	RA (mg/g)	Eupatorin (mg/g)	TMF (mg/g)	Sinensetin (mg/g)
Crude OS	196.14 ± 0.33	168.54 ± 0.28	11.38 ± 0.28	18.87 ± 0.12
F1	65.62 ± 0.44			
F2	148.62 ± 0.46			
F3	2.42 ± 0.22	30.02 ± 0.33		
F4		114.24 ± 0.40	89.88 ± 0.45	45.88 ± 0.40
F5		23.03 ± 0.16	27.18 ± 0.54	
F6		21.32 ± 0.15		

**Table 6:** IC_50_ values for 2,2′-Azino-bis(3-Ethylbenzothiazoline-6-sulphonic acid) (ABTS) and 2,2-Diphenyl-1-picrylhydrazyl (DPPH) radical scavenging.

Samples	IC_50_ Value (µg/mL)	
	ABTS	DPPH
Crude	67.31	23.03
F1	38.39	15.86
F2	11.29	5.975
F3	24.84	9.958
F4	86.99	67.10
F5	N/A	N/A
F6	N/A	N/A
Trolox	3.407	-
Ascorbic acid		3.047

**Table 7:** IC_50_ values of crude OS and its fractions on HSF 1184 and DU145. IC_50_ represents the half maximal inhibitory concentration to determine the potency of the samples in inhibiting a biological function.

Samples	IC_50_ (µg/mL)	
	Normal Cell Line (HSF 1184)	Prostate Cancer Cell Line (DU145)
Crude OS	169.4	26.99
F1	Not converge (>1000)	101.8
F2	695.1	43.01
F3	175.9	229.8
F4	14.47	12.38
F5	62.07	26.63
F6	20.60	18.68

**Table 8:** Gradient elution system for high-performance liquid chromatography (HPLC) analysis.

Time	Flow Rate (mL/min)	Solvent Ratio	
		A (0.1% Formic Acid)	B (Acetonitrile)
0	1	85	15
1	1	85	15
12	1	35	65
15	1	85	15
18	1	85	15

**Table 9:** Level of extraction parameters.

Independent Variables	Level	
	−1	+1
Extraction time (minutes)	15	25
Amplitude (A)	40	80
Percentage of solvent (%)	60	80

**Table 10:** Elution solvent ratio (water–acetonitrile).

Fraction	Solvent Ratio (%)	
	Water	Acetonitrile
1	100	0
2	80	20
3	60	40
4	40	60
5	20	80
6	0	100
